# Dissemination of KPC-2-producing carbapenem-resistant *Klebsiella pneumoniae* ST792 in Southern China

**DOI:** 10.3389/fmicb.2025.1580739

**Published:** 2025-06-06

**Authors:** Cailing Wan, Meilan Li, Haojin Gao, Peiyao Zhou, Bingjie Wang, Junhong Shi, Li Shen, Weihua Han, Xinru Yuan, Jianbo Lv, Yu Huang, Ying Zhou, Fangyou Yu

**Affiliations:** ^1^Department of Clinical Laboratory, Shanghai Pulmonary Hospital, School of Medicine, Tongji University, Shanghai, China; ^2^School of Public Health, Jiangxi Medical College, Nanchang University, Nanchang, China; ^3^Department of Laboratory Medicine, The First Affiliated Hospital of Wenzhou Medical University, Wenzhou, China

**Keywords:** carbapenem-resistant *Klebsiella pneumoniae*, molecular epidemiology, dissemination, ST792, whole-genome sequencing

## Abstract

**Background:**

The global emergence of carbapenem-resistant *Klebsiella pneumoniae* (CRKP) has become a critical public health threat. However, the epidemiological significance of certain sequence types (STs) remains underappreciated. Among these, ST792—a lineage rarely documented in global surveillance studies—has recently emerged as a concerning threat in southern China. In this study, we characterized the epidemiological features and antimicrobial resistance mechanisms of CRKP ST792 isolates collected during a dissemination in a hospital in southern China.

**Methods:**

Seven separate clinical isolates were collected from hospitalized patients between January 2021 and March 2022. Bacterial isolates were identified, and antimicrobial susceptibility testing was conducted using the VITEK-2 compact automated system. Whole-genome sequencing (WGS) was performed on all seven isolates to confirm the presence of resistance genes. Additionally, a representative strain (G5) was selected for in-depth genomic characterization using long-read sequencing to analyze its genetic features and mobile genetic elements. Conjugation experiments were conducted to assess the transferability of the resistance plasmids.

**Results:**

All isolated strains were identified as ST792-type CRKP carrying bla_KPC − 2_ through whole-genome sequencing. The strains harbored additional resistance genes including bla_SHV − 148_, bla_CTX − M−3_, bla_TEM − 1B_, qnrS1, OqxA and OqxB. Genomic characterization of representative strain G5 revealed a circular chromosome and three resistance plasmids. The bla_KPC − 2_ gene was located on a 102,257 bp IncFIB(pQil) plasmid with a Tn3-TnpR-ISKpn27-ISKpn28-bla_KPC − 2_-ISKpn6 genetic structure. Conjugation experiments demonstrated successful transfer of two accessory plasmids (p[G5]-2 and p[G5]-3) to Escherichia coli EC600, confirming their mobility and potential role in resistance gene dissemination.

**Conclusion:**

This study characterizes the nosocomial dissemination of KPC-2-producing *K. pneumoniae* ST792 strains, elucidating their antimicrobial resistance patterns and plasmid-mediated transmission mechanisms to inform infection control strategies. The urgent need for enhanced surveillance and strict implementation of infection control measures is underscored to mitigate the spread of hospital-acquired multidrug-resistant pathogens.

## Introduction

*Klebsiella pneumoniae*, a Gram-negative opportunistic pathogen of the Enterbacteriaceae, is a leading cause of nosocomial infections including pneumonia, soft tissue infection, urinary tract infection and septicemia (Podschun and Ullmann, 1998). Carbapenems, along with other β-lactam antibiotics, serve as first-line therapeutics for severe *K. pneumoniae* infections and represent last-resort options for multidrug-resistant (MDR) cases. Since the first reported isolation of carbapenem-resistant *K. pneumoniae* (CRKP) in the 1990s (MacKenzie et al., 1997), these resistant pathogens have rapidly disseminated globally (Clancy et al., 2013; Xu et al., 2017; Zheng et al., 2018). CRKP infections pose a critical public health challenge, associated with significantly higher mortality rates particularly among immunocompromised patients and those in intensive care settings (Karampatakis et al., 2023).

The antimicrobial resistance mechanisms of CRKP are mediated through three principal pathways: (1) enzymatic inactivation via β-lactamase production, including extended-spectrum β-lactamases (ESBLs) and carbapenemases; (2) enhanced efflux pump activity; and (3) structural modifications of outer membrane proteins (Pu et al., 2023). Among these, carbapenemase production has been established as the predominant resistance mechanism in CRKP clinical isolates (Gandra and Burnham, 2020; Karaiskos et al., 2021). Carbapenemases are phylogenetically classified into three molecular classes (A, B, and D). Among class A carbapenemases, KPC-type enzymes represent the most clinically significant group (Zhang et al., 2017), with phylogenetic analyses identifying numerous variants (KPC-2 through KPC-157) across different geographical regions (Naas et al., 2017).

The KPC enzyme was first identified in a clinical isolate of *K. pneumoniae* from North Carolina, USA, in 1996 (Seki et al., 2011). Since its initial detection, the global prevalence of KPC-producing strains has increased dramatically (Yigit et al., 2001; Nordmann et al., 2009). While numerous KPC variants have been characterized, epidemiological surveillance indicates that KPC-2 and KPC-3 remain the predominant variants worldwide (Walther-Rasmussen and Høiby, 2007). Geographic distribution patterns reveal distinct genotype predominance: bla_KPC − 3_ represents the major genotype in the United States (Sader et al., 2017), whereas bla_KPC − 2_ dominates in China (Zhang et al., 2018). The spread of bla_KPC_ involves clonal expansion, horizontal transfer, and dissemination via plasmids. In healthcare settings, the emergence of KPC-producing *K. pneumoniae* has been largely driven by specific epidemic clones (Cuzon et al., 2010). ST258 prevails as the dominant sequence type in the United States (Kitchel et al., 2009), while ST11 clones account for most clinical isolates in China (Qi et al., 2011). These high-risk clones have become a major focus of antimicrobial resistance research due to their rapid global dissemination and association with treatment failures (Dong et al., 2018; Munoz-Price et al., 2013).

*K. pneumoniae* ST792 represents a distinct sequence type within the multilocus sequence typing (MLST) classification system. While demonstrating lower global prevalence compared to pandemic clones like ST258 and ST11, ST792 has been identified across multiple geographical regions including Europe (Jati et al., 2023), Asia (Anuar et al., 2024), North America (Wang et al., 2013), and the Middle East (Eltai et al., 2020). Clinical isolates are primarily recovered from both healthcare-associated and community-acquired infections, with specimen sources encompassing rectal colonization, urinary tract infections, wound infections, and respiratory tract infections. Most ST792 strains exhibit MDR without carbapenemase production, as exemplified by a United States isolate carrying CTX-M-2, SHV-11 and TEM-1 (Wang et al., 2013), and an ESBL-producing strain from Malaysia (Anuar et al., 2024). However, rare cases of carbapenem-resistant ST792 have been reported, such as a KPC-2-producing ST792 isolate documented in Singapore (Octavia et al., 2019). To date, few studies have systematically investigated the nosocomial transmission dynamics of ST792 CRKP carrying KPC-2 or the mobility of its drug-resistant plasmids in clinical settings.

In this study, we characterized a hospital-based dissemination of ST792 CRKP harboring bla_KPC − 2_ between intensive care and neurosurgery units. Through whole-genome sequencing, we elucidated the molecular resistance mechanisms and transmission pathways to guide clinical anti-infective treatment and infection control measures against resistant strain transmission.

## Materials and methods

### Bacterial isolation and clinical information

Between January 2021 and March 2022, seven nonrepeated CRKP isolates were collected from a tertiary hospital in Ganzhou, Jiangxi Province, China. All strains were isolated from patients aged >45 years with severe underlying conditions. Isolates G5, G9, G10 and G18 were obtained from sputum specimens, while G11, G28 and G34 were recovered from bronchoalveolar lavage fluid. The patients exhibited diverse clinical presentations: the G5-infected individual from the intensive care unit (ICU) was diagnosed with skull fracture; G9, G28, and G34 were isolated from cardiac care unit (CCU) patients with hydrocephalus, cerebral infarction, and acute myocardial infarction, respectively; G10 and G18 originated from Neurosurgery Department Area two cases with left basal ganglia hemorrhage and cerebral hemorrhage; and G11 was cultured from a high-dependency unit (HDU) patient with cerebral hemorrhage. All isolates demonstrated carbapenem-resistant phenotypes by VITEK-2 Compact system analysis, using Escherichia coli ATCC 25922 as the quality control strain for antimicrobial susceptibility testing (AST).

### Antimicrobial susceptibility testing

The minimum inhibitory concentrations (MICs) of Amoxicillin/Clavulanic acid, Piperacillin/Tazobactam, Cefuroxime, Cefuroxime axetil, Cefoxitin, Ceftazidime, Ceftriaxone, Cefoperazone/Sulbactam, Cefepime, Ertapenem, Imipenem, Amikacin, Levofloxacin, Trimethoprim/Sulfamethoxazole were determined using the VITEK 2 Compact system (bioMérieux, France), with results interpreted according to Clinical and Laboratory Standards Institute (CLSI2022-M100-ED31) guidelines. For tigecycline, MIC was assessed via broth dilution in Mueller-Hinton broth, with interpretation based on European Committee on Antimicrobial Susceptibility Testing (EUCAST 2022) breakpoint. Quality control was performed using Escherichia coli ATCC 25922 for all antimicrobial susceptibility testing procedures to ensure accuracy and reproducibility.

### Whole genome sequencing and bioinformatics analysis

Genomic DNA was extracted using a commercial genomic DNA extraction kit (Qiagen, Germany) following the manufacturer's protocol. Whole genome sequencing was performed using both short-read and long-read sequencing technologies. For short-read sequencing, paired-end libraries (2 × 125 bp) were prepared and sequenced on the Illumina NovaSeq 6000 platform. Long-read sequencing was conducted using the PacBio Sequel platform with a 10-kb insert library. Raw sequencing data were processed by removing adapter sequences using AdapterRemoval (Lindgreen, 2012) and quality filtering with SOAPec (Luo et al., 2012). High-quality reads were *de novo* assembled into contigs and scaffolds using SPAdes v3.12 (Bankevich et al., 2012) and A5-miseq v20160825 (Coil et al., 2015). PacBio long reads were assembled using CANU v.1.7.1 (Koren et al., 2017). The final genome sequence was generated by integrating both Illumina and PacBio assemblies, followed by error correction using Pilon v1.23. The assembled genome was annotated using Prokka v1.14.6 (Seemann, 2014) and the RAST server v2.0 (Brettin et al., 2015). Multi-locus sequence typing (MLST) was performed using MLST v2.23.0 (https://cge.cbs.dtu.dk/services/MLST/), while capsular serotyping and virulence factor identification were conducted using Kleborate v2.0.0 (https://github.com/katholt/Kleborate). Antibiotic resistance genes were identified using ResFinder 4.1 (https://cge.cbs.dtu.dk/services/ResFinder/) with default parameters. Core genome SNPs were identified through stringent filtering excluding: (i) low-complexity regions (<200 bp), (ii) 100-bp windows with <50% column consistency, and (iii) 100-bp windows containing >20 indels. Maximum-likelihood trees were reconstructed using RAxML-NG (GTR+GAMMA model, 1,000 bootstraps) based on the filtered core SNP alignment, with pairwise SNP distances calculated using MEGA-CC. Plasmid analysis included conjugation potential prediction using oriTfinder (http://bioinfo-mml.sjtu.edu.cn/oriTfinder/) and visualization with Proksee v1.2.0 (https://proksee.ca/). The genetic environment surrounding bla_KPC − 2_ in plasmid p[G5]-2 was compared with reference sequence pKPHS2_KPC-2 using Easyfig v2.2.3. A Minimum Spanning Tree (MST) was constructed using PhyloViz 2.0 based on over 8,000 *K. pneumoniae* STs from the Pasteur MLST database (PubMLST) (Institute Pasteur, 2025). The complete genomic sequence of G5 has been deposited in GenBank under accession no. CP170118. We also deposited the raw reads of the genomes of the remaining six strains we sequenced in GenBank (Bioproject accession no. PRJNA1253637).

### Conjugation experiments

To assess the transferability of resistance plasmids from *K. pneumoniae* G5, a conjugation assay was performed using rifampicin-resistant E. coli EC600 as the recipient strain. Donor and recipient cultures were grown to logarithmic phase, mixed at a 1:9 ratio, pelleted by centrifugation (8,000 × g, 2 min), and resuspended in 20 μL of LB broth. After 24 h of incubation at 37°C, transconjugants harboring p[G5]-2 were selected on LB agar supplemented with rifampicin (200 μg/mL) and meropenem (1 μg/mL), while transconjugants carrying p[G5]-3 were selected using rifampicin (200 μg/mL) and ciprofloxacin (10 μg/mL). Conjugation frequency was calculated as the number of transconjugants per donor cell. Successful plasmid transfer was confirmed via PCR amplification of bla_KPC − 2_ and qnrS1 using primers listed in Table 1. Additionally, the MICs of transconjugants were determined using the VITEK-2 Compact system to evaluate phenotypic resistance profiles.

**Table 1:** Oligonucleotides for PCR.

**Name**	**Sequence**
bla_KPC − 2_-F	TCGCTAAACTCGAACAGG
bla_KPC − 2_-R	TTACTGCCCGTTGACGCCCAATCC
qnrS1-F	ACAATCATACATATCGGC
qnrS1-R	TTTTTCTAAACAAACCCT

## Results

### The clinical features of ST792 *Klebsiella pneumoniae*

**Figure 1:**
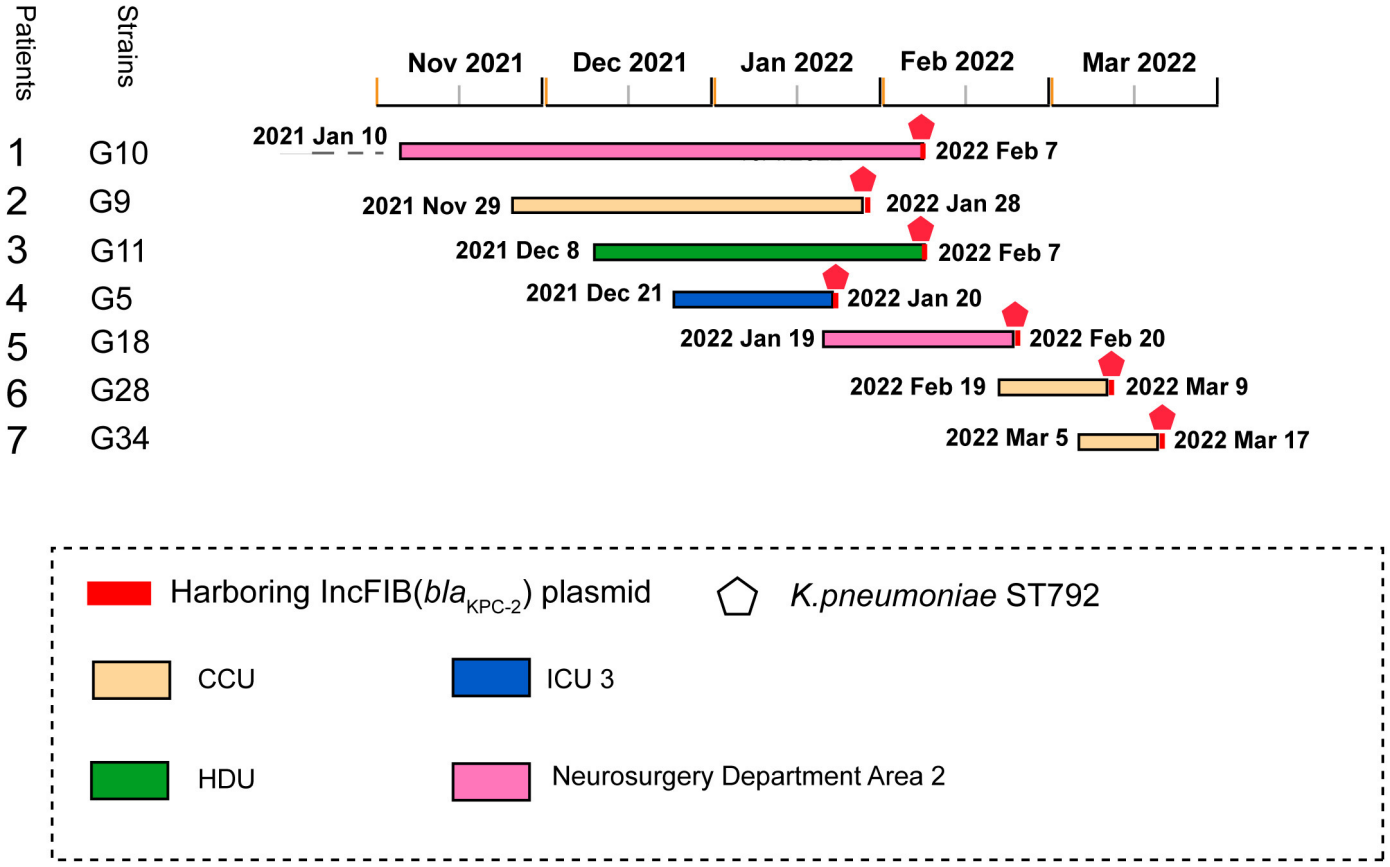
Dissemination timeline of KPC-2-producing *K. pneumoniae* infection at a tertiary hospital in Ganzhou, Jiangxi Province, China. CCU, cardiac care unit; HDU, high dependency unit; ICU, intensive care unit.

From January 10, 2021, to March 17, 2022, we conducted whole-genome sequencing on all CRKP isolates collected from a tertiary hospital in southern China. Genomic analysis revealed seven KPC-2-producing ST792 *K. pneumoniae* strains isolated from distinct patients. The uncommon detection frequency of ST792 *K. pneumoniae* prompted further analysis of its epidemiological characteristics. These strains were isolated from respiratory specimens, including sputum (*n* = 4) and bronchoalveolar lavage fluid (*n* = 3). The first bla_KPC − 2_-positive isolate was detected in January 2022 from a patient admitted to the ICU following a skull fracture. A CRKP strain was recovered from the patient's sputum after 30 days of hospitalization. Subsequent CRKP isolates (*n* = 3) were identified in multiple hospital wards, including the CCU (*n* = 1), HDU (*n* = 1), ICU (*n* = 1), and Neurosurgery Department Area 2 (*n* = 2) (Figure 1).

The median patient age was 67 years (range: 48–92), with a male predominance (57.1%). All bla_KPC − 2_-positive isolates were detected ≥10 days post-admission (range: 12–393 days). Underlying conditions included cerebral hemorrhage (*n* = 3), hydrocephalus (*n* = 1), cerebral infarction (*n* = 1), skull fracture (*n* = 1), and acute myocardial infarction (*n* = 1). Of these patients, 57.1% underwent surgical procedures, and all received mechanical ventilation and carbapenem antibiotics prior to CRKP detection (Table 2).

**Table 2:** Clinical characteristics of KPC-2-producing *K. pneumoniae*.

**Patients**	**Species**	**Age**	**Gender**	**Isolates**	**Time of admission**	**Time of Isolation**	**Source**	**Ward**	**Major diagnosis**
1	*K. pneumoniae*	48	Male	G5	2021/12/21	2022/1/20	Sputum	ICU 3	Fracture of skull
2	*K. pneumoniae*	73	Female	G9	2021/11/29	2022/1/28	Sputum	CCU	Hydrocephalus
3	*K. pneumoniae*	67	Male	G10	2021/1/10	2022/2/7	Sputum	Neurosurgery Department Area 2	Left basal ganglia cerebral hemorrhage
4	*K. pneumoniae*	73	Female	G11	2021/12/8	2022/2/7	Alveolar lavage fluid	HDU	Cerebral hemorrhage
5	*K. pneumoniae*	55	Male	G18	2022/1/19	2022/2/22	Sputum	Neurosurgery Department Area 2	Cerebral hemorrhage
6	*K. pneumoniae*	92	Female	G28	2022/2/19	2022/3/9	Alveolar lavage fluid	CCU	Cerebral infarction
7	*K. pneumoniae*	63	Male	G34	2022/3/5	2022/3/17	Alveolar lavage fluid	CCU	Acute myocardial infarction

### Antimicrobial susceptibility and resistance genes

During antimicrobial susceptibility testing of the seven ST792 CRKP isolates against 15 antibiotics, all strains demonstrated resistance to amoxicillin/clavulanic acid, piperacillin/tazobactam, cefuroxime, cefuroxime axetil, cefoxitin, ceftazidime, ceftriaxone, cefoperazone/sulbactam, cefepime, ertapenem, imipenem, and levofloxacin, while remaining susceptible to amikacin and trimethoprim/sulfamethoxazole (Table 3). Genomic analysis revealed that all CRKP isolates harbored multiple resistance genes, including bla_KPC − 2_, qnrS1, bla_CTX − M−3_, bla_SHV − 1_, and bla_TEM − 1B_. The presence of qnrS1 correlated with ciprofloxacin resistance, bla_KPC − 2_ mediated resistance to cephalosporins and carbapenems, bla_SHV − 1_ and bla_TEM − 1B_ conferred ampicillin resistance, and bla_CTX − M−3_ was associated with resistance to both ampicillin and ceftriaxone, collectively explaining the observed multidrug-resistant phenotype.

**Table 3:** Antimicrobial drug susceptibility profiles of KPC-2-producing *K. pneumoniae*.

**Antibiotics**	**MIC(**μ**g/mL)**						
	**G5**	**G9**	**G10**	**G11**	**G18**	**G28**	**G34**
AMC	≥32(R)	≥32(R)	≥32(R)	≥32(R)	≥32(R)	≥32(R)	≥32(R)
TZP	≥128(R)	≥128(R)	≥128(R)	≥128(R)	≥128(R)	≥128(R)	≥128(R)
CXM	≥64(R)	≥64(R)	≥64(R)	≥64(R)	≥64(R)	≥64(R)	≥64(R)
CAX	≥64(R)	≥64(R)	≥64(R)	≥64(R)	≥64(R)	≥64(R)	≥64(R)
FOX	8(R)	16(R)	8(R)	16(R)	16(R)	16(R)	16(R)
CAZ	32(R)	≥32(R)	≥32(R)	≥32(R)	≥32(R)	≥32(R)	≥32(R)
CRO	≥64(R)	≥64(R)	≥64(R)	≥64(R)	≥64(R)	≥64(R)	≥64(R)
CFP/SU	≥64(R)	≥64(R)	≥64(R)	≥64(R)	≥64(R)	≥64(R)	≥64(R)
FEP	≥32(R)	≥32(R)	≥32(R)	≥32(R)	≥32(R)	≥32(R)	≥32(R)
ETP	2(R)	2(R)	2(R)	2(R)	2(R)	2(R)	4(R)
MEM	8(R)	≥16(R)	8(R)	≥16(R)	8(R)	8(R)	≥16(R)
AMK	≤ 2(S)	4(S)	≤ 2(S)	≤ 2(S)	≤ 2(S)	≤ 2(S)	≤ 2(S)
LVX	4(R)	4(R)	4(R)	4(R)	4(R)	4(R)	4(R)
TGC	4(I)	4(I)	4(I)	4(I)	4(I)	2(S)	4(I)
SXT	≤ 20(S)	≤ 20(S)	≤ 20(S)	≤ 20(S)	≤ 20(S)	≤ 20(S)	≤ 20(S)

MIC, minimum inhibitory concentration; AMC, Amoxicillin/Clavulanic; TZP, Piperacillin/Tazobactam; CXM, Cefuroxime; CAX, Cefuroxime Axetil; FOX, Cefoxitin; CAZ, Ceftazidime; CRO, Ceftriaxone; CFP/SU, Cefoperazone/Sulbactam; FEP, Cefepime; ETP, Ertapenem; IMP, Imipenem; AMK, Amikacin; LVX, Levofloxacin; TGC, Tigecycline; SXT, Trimethoprim/Sulfamethoxazole.

### Molecular epidemiology

**Figure 2:**
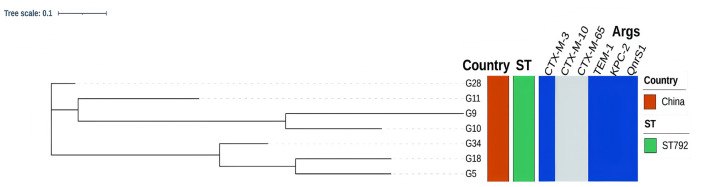
Distribution of resistance genes of ST792 CRKP isolates. The phylogenetic showed that the ST792 CRKP isolates were divided into three subgroups. Resistance genes are labeled in blue, with blank spaces indicating gene absence.

MLST analysis of 7 housekeeping genes of *K. pneumoniae* showed that all 7 CRKP isolates belonged to the ST792 strain. For the seven isolates, we constructed a phylogenetic using maximum likelihood based on the core genomic SNPs, and the results were divided into three subgroups, G11, G9 and G10 with highly homology, G34, G18 and G5 with high homology, G28 is a separate subgroup (Figure 2, Supplementary Table 2). To trace phylogenetic origins and characterize population structure, we performed comparative genomic analysis of our ST792 isolates with publicly available ST792 strains from National Center for Biotechnology Information (NCBI) RefSeq database (https://www.ncbi.nlm.nih.gov/refseq [accessed May 10, 2025]). Our analysis revealed that the seven ST792 isolates from this study formed a distinct cluster, evolutionarily distant from four other ST792 strains from different countries, suggesting independent origins. Notably, all seven isolates in our study carried the bla_KPC − 2_ gene and were carbapenem-resistant, whereas the four comparator strains were susceptible to carbapenems and lacked this resistance determinant (Supplementary Figure 1, Supplementary Table 3).

### MLST-based minimum spanning tree construction

The MST analysis revealed that ST792 is directly connected to its two closest phylogenetic relatives—ST1373 and ST5091—which differ by two housekeeping gene loci (infB and pgi) and one locus (tonB), respectively (Supplementary Figure 2, Supplementary Table 1). Notably, both ST1373 and ST5091 are rare STs in *K. pneumoniae*, suggesting potential niche adaptation or limited transmission of these closely related variants.

### The characteristic analysis of G5 plasmids

**Figure 3:**
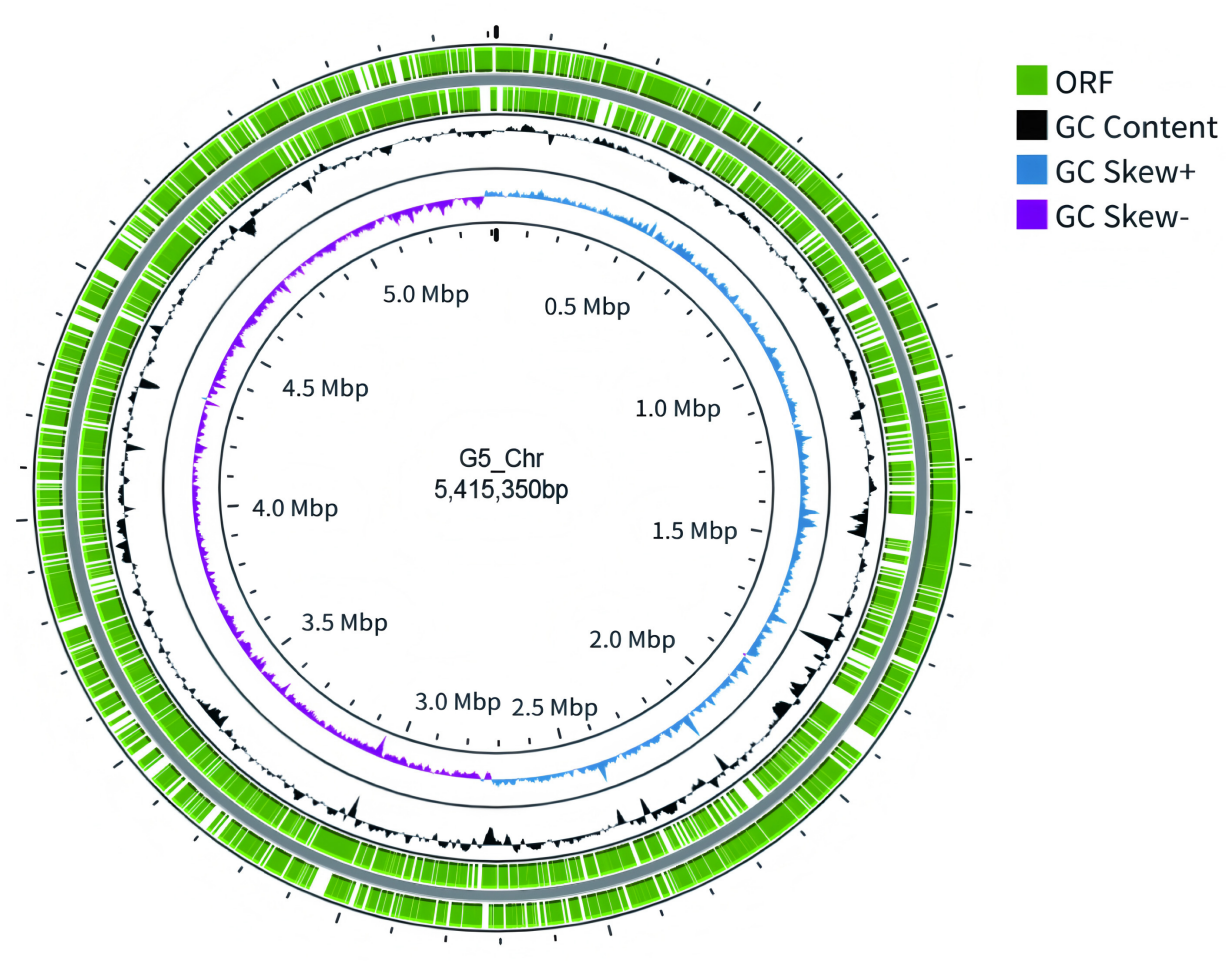
Schematic circular genome of G5. Moving from the outermost to the innermost circles: the first and second circles highlight the coding sequences on the positive and negative strands, respectively; the third circle charts the GC content, with heightened levels indicated on the exterior, suggesting these areas have a higher GC content than the genome average; the fourth circle depicts the GC skew values.

Given identical genomic profiles among all seven ST792 isolates by Illumina sequencing, we selected G5 (the first isolated strain) for PacBio long-read sequencing to elucidate resistance gene contexts and transmission-relevant genomic features. The draft genome of *K. pneumoniae* G5 comprises 5,744,391 bp with an N50 of 5,415,350 bp and a guanine and cytosine content of 57%. The genome consists of a circular chromosome of 5,415,350 bp and four plasmids: p[G5]-1 (146,367 bp), p[G5]-2 (102,257 bp), p[G5]-3 (71,123 bp), p[G5]-4 (9,294 bp). Prokka annotation identified 5,067 protein-coding genes and 111 RNA genes on the chromosome (Figure 3).

**Figure 4:**
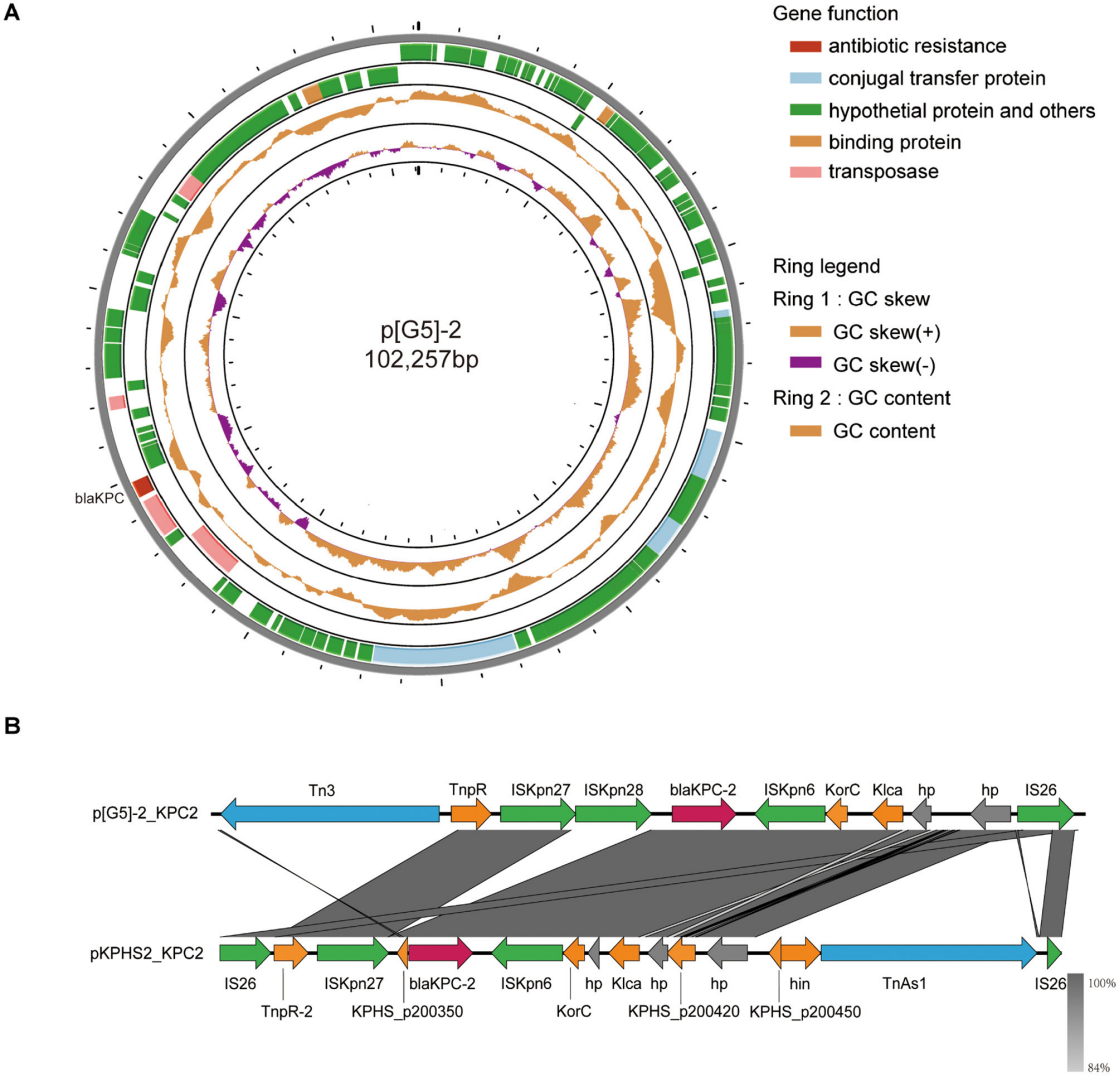
**(A)** Circular map of p[G5]-2 of *K. pneumoniae* G5. **(B)** Linear comparison of the bla_KPC − 2_ region of p[G5]-2 and plasmid pKPHS2. Open reading frames are portrayed by arrows and are depicted in different colors on the basis of their predicted gene functions. Red arrows indicate resistance genes, blue arrows indicate transposon, green arrows indicate insertion sequence, orange arrows represent the backbone genes of the plasmid, and gray arrows indicate hypothetical protein.

Whole-genome sequencing analysis identified three resistance plasmids in this isolate (Table 4), with particular attention given to bla_KPC − 2_, a critical determinant of carbapenem resistance. The plasmid p[G5]-2, which carries the carbapenem resistance gene bla_KPC − 2_, belongs to the IncFIB(pQil) incompatibility group. A schematic representation of the genetic environment of the bla_KPC − 2_ genes on the p[G5]-2 is shown in Figure 4A. The analysis further revealed that plasmids p[G5]-1 and p[G5]-3 carry multiple resistance determinants contributing to the strain's multidrug-resistant phenotype. Specifically, p[G5]-1 harbors extended-spectrum β-lactamase genes (bla_CTX − M−3_ and bla_TEM − 1B_), while p[G5]-3 contains both these β-lactamase genes along with the fluoroquinolone resistance determinant qnrS1.

**Table 4:** General features and antimicrobial resistance genes of plasmids in *K. pneumoniae* G5.

	**G5**			
**Characteristics**	**p[G5]-1**	**p[G5]-2**	**p[G5]-3**	**p[G5]-4**
Accession no	CP170119	CP170120	CP170121	CP170122
Length (bp)	1,46,367	1,02,257	71,123	9,294
GC content (%)	51	53	52	55
No. of ORF^a^	310	247	171	25
Incompability group	IncFIB/IncFII	IncFIB	/^b^	ColRNAI
**Conjugal ability**				
*OriT* (start...stop) (bp)	45331.0.45380	21086.0.21135	63518..63599	/
Relaxase (start...stop) (bp)	010658.0.15916	048272.0.53530	063955..65883	/
T4CP (start...stop) (bp)	015916.0.18225	45954.0.48272	023520..25712	/
T4SS (start...stop) (bp)	9843.0.45932	20528.0.54338	619..25712	/
Resistant genes	*bla*_CTX − M−3_	*bla*_KPC − 2_	*bla*_CTX − M−3_	/
	*bla*_TEM − 1B_		*bla*_TEM − 1B_	/
			*qnrS1*	/
Virulence factors	/	/	/	/

^a^ORF, open reading frame.

^b^/, no such information.

### Genetic environment of bla_*kpc*−2_ gene

The resistance plasmid p[G5]-2 exhibits a content of 53% and carries a single carbapenemase resistance gene, bla_KPC − 2_. This gene is located within a conserved mobile genetic element structure composed of Tn3-tnpR-ISKpn27-ISKpn28-bla_KPC_-2-ISKpn6, representing a characteristic Non-Tn4401 Element-KPC type 1 (NTEKPC-1) structure where KPC is associated with a non-Tn4401 transposon.

Comparative analysis with the reference plasmid pKPHS2 (CP003224.1), isolated from a Shanghai clinical strain, revealed that p[G5]-2 shares over 80% nucleotide identity in the backbone region. A distinctive feature of p[G5]-2 is the presence of an ISKpn28 insertion immediately upstream of bla_KPC − 2_ (Figure 4B), which is absent in pKPHS2. This insertion may potentially enhance the plasmid's conjugative transfer capacity, as IS elements are known to promote horizontal gene transfer through recombination and mobilization of adjacent genetic material (Johnson and Grossman, 2015).

### Conjugation experiments

We conducted conjugation experiments to assess the transferability of three resistant plasmids from strain G5 using E. coli EC600 as the recipient. The assays demonstrated successful horizontal transfer of p[G5]-2 (carrying bla_KPC − 2_) and p[G5]-3 (harboring bla_CTX − M−3_, bla_TEM − 1B_ and qnrS1) to recipient strains, with transfer frequencies ranging from 3.13 × 10^−8^ to 1.82 × 10^−7^ and 2.85 × 10^−4^ to 1.39 × 10^−3^, respectively. Antimicrobial susceptibility testing confirmed the acquisition of carbapenem and fluoroquinolone resistance phenotypes in the transconjugants (Table 5). Notably, because the resistance phenotype mediated by p[G5]-1 was phenotypically indistinguishable from those encoded by p[G5]-3, we were unable to specifically assess p[G5]-1′s conjugation frequency using conjugation experiments.

**Table 5:** Comparison of donors and transconjugants' minimal inhibitory concentrations.

**Antibiotics**	**MIC(**μ**g/mL)**			
	**G5**	**EC600**	**p[G5]-2-EC600(bla**_KPC − 2_ **plasmid)**	**p[G5]-3-EC600(bla**_CTX − M−3_**, bla**_TEM − 1B_ **and qnrS1 plasmid)**
AMC	≥32(R)	4(S)	≥32(R)	16(I)
TZP	≥128(R)	≤ 4(S)	≥128(R)	≤ 4(S)
CXM	≥64(R)	8(S)	≥64(R)	≥64(R)
CAX	≥64(R)	8(I)	≥64(R)	≥64(R)
FOX	8(R)	8(S)	16(R)	8(S)
CAZ	32(R)	0.5(S)	16(R)	4(S)
CRO	≥64(R)	≤ 0.25(S)	32(R)	≥64(R)
CFP/SU	≥64(R)	≤ 8(S)	32(R)	16(S)
FEP	≥32(R)	≤ 0.12(S)	2(S)	16(R)
ETP	2(R)	≤ 0.12(S)	1(I)	≤ 0.12(S)
MEM	8(R)	≤ 0.25(S)	8(R)	≤ 0.25(S)
AMK	≤ 2(S)	≤ 2(S)	≤ 2(S)	≤ 2(S)
LVX	4(R)	0.5(S)	0.5(S)	4(R)
TGC	4(I)	≤ 0.5(S)	≤ 0.5(S)	≤ 0.5(S)
SXT	≤ 20(S)	≤ 20(S)	≤ 20(S)	≤ 20(S)

G5 is the donor bacteria; EC600 is the recipient.

## Discussion

From January 2021 to March 2022, seven CRKP isolates of were identified across ICU, CCU, HDU and neurosurgery units in our hospital. Through phylogenetic analysis, it was discovered that the ST792 CRKP isolates could be categorized into three subgroups, indicating possible clonal transmission among them. However, phylogenetic analysis revealed significant genetic divergence between these seven *K. pneumoniae* strains and the four ST792 reference strains available in the NCBI database, demonstrating no evident evolutionary relationship between these clusters. The MST analysis revealed that ST792 occupies a phylogenetically distinct position within the *K. pneumoniae* sequence type network, forming a relatively independent branch, meanwhile demonstrating closest phylogenetic relationships with two other rare ST types (ST1373 and ST5091). In contrast to ST11 (Qi et al., 2011) - the dominant epidemic clone in China, *K. pneumoniae* ST792 has been reported only as sporadic cases internationally, with documented occurrences in the Netherlands (Jati et al., 2023) and USA (Wang et al., 2013). To the best of our knowledge, this represents the first comprehensive characterization of *K. pneumoniae* ST792′s genomic features.

All seven CRKP strains exhibited uniform resistance to carbapenems, extended-spectrum cephalosporins, and fluoroquinolones, while maintaining susceptibility to amikacin and Trimethoprim/sulfamethoxazole. Whole-genome analysis identified a conserved repertoire of resistance determinants, including bla_KPC − 2_, bla_CTX − M−3_, bla_TEM − 1B_, bla_SHV − 1_, and qnrS1, with each isolate harboring three or more resistance genes. The observed multidrug-resistant phenotype likely results from synergistic interactions between these co-localized resistance determinants.

The bla_KPC_ genes, located on transmittable plasmids, have the potential for rapid dissemination, making infections caused by KPC-producing *K. pneumoniae* difficult to control once they become prevalent (Smith Moland et al., 2003; Woodford et al., 2004; Bradford et al., 2004). The conjugation experiment also showed that the resistance genes of G5 isolate can be successfully transferred to the transconjugants, confirming that the resistance genes could be transferred horizontally between bacteria through plasmids, which accelerates the spread process between bacteria.

All ST792 CRKP isolates carrying bla_KPC_ have been identified through whole genome sequencing analyses, with bla_KPC_ commonly found on various plasmids types, including IncF, IncI, IncA/C, IncN, IncX, IncR, IncP, IncU, IncW, IncL/M, and ColE plasmids (Chen et al., 2014). Based on differences between plasmid replicons, bla_KPC_ appears to predominantly reside in IncF plasmid (Chen et al., 2014), which is consistent with our findings. We further analyzed the genetic environment of the resistance genes. The p[G5]-2 plasmid of strain G5 harbors only one carbapenem-resistance gene, bla_KPC − 2_. This gene resides within an IS element cluster/transposon structure designated as “Tn3-tnpR-ISKpn27-ISKpn28-bla_KPC − 2_-ISKpn6”. This arrangement exhibits similarity to the conventional NTEKPC-I structure, where the bla_KPC − 2_ gene is located on a non-Tn4401 transposon, differing from the typical Tn4401 transposon, as demonstrated in the literature (Cuzon et al., 2011). Notably, p[G5]-2 contains an additional ISKpn28 insertion compared to the reference plasmid pKPHS2, suggesting potentially enhanced horizontal transfer capacity (De Souza et al., 2019).

This study documents a nosocomial dissemination of KPC-2-producing *K. pneumoniae* ST792 in a tertiary hospital in Ganzhou, China, characterized through whole-genome sequencing analysis. To our knowledge, this represents the first report detailing the genetic context of resistance determinants and plasmid-mediated transfer mechanisms in the ST792 lineage. The analyzed ST792 CRKP isolates demonstrated a concerning multidrug resistance profile, exhibiting high-level resistance to most clinically relevant antibiotics. This phenotype correlated with the co-occurrence of a plasmid-encoded quinolone resistance determinant (qnrS1) and multiple β-lactamase genes (bla_KPC − 2_, bla_CTX − M−3_, and bla_TEM − 1B_), suggesting synergistic contributions to the observed resistance patterns. Additionally, phylogenetic analysis suggests that ST792 CRKP may have spread widely in localized areas. Therefore, ongoing surveillance in hospital wards is clearly needed to mitigate the colonization and proliferation of these resistant bacteria.

## Data Availability

We also deposited the raw reads of the genomes of the remaining six strains we sequenced in GenBank (Bioproject accession no. PRJNA1253637).

## References

[B1] Anuar N. S. Hazman H. Jeyakumar S. R. Desa M. N. M. Saidi H. I. Sulaiman N.. (2024). Occurrence of K1 and K2 serotypes and genotypic characteristics of extended spectrum β-lactamases-producing *Klebsiella pneumoniae* isolated from selected hospitals in Malaysia. Asian Pac. J. Trop. Med. 17, 30–38. 10.4103/apjtm.apjtm_303_23

[B2] Bankevich A. Nurk S. Antipov D. Gurevich A. A. Dvorkin M. Kulikov A. S.. (2012). SPAdes: a new genome assembly algorithm and its applications to single-cell sequencing. J. Comput. Biol. 19, 455–477. 10.1089/cmb.2012.0021 22506599 PMC3342519

[B3] Bradford P. A. Bratu S. Urban C. Visalli M. Mariano N. Landman D.. (2004). Emergence of carbapenem-resistant *Klebsiella* species possessing the class A carbapenem-hydrolyzing KPC-2 and inhibitor-resistant TEM-30 beta-lactamases in New York City. Clin. Infect. Dis. 39, 55–60. 10.1086/421495 15206053

[B4] Brettin T. Davis J. J. Disz T. Edwards R. A. Gerdes S. Olsen G. J.. (2015). RASTtk: a modular and extensible implementation of the RAST algorithm for building custom annotation pipelines and annotating batches of genomes. Sci. Rep. 5:8365. 10.1038/srep08365 25666585 PMC4322359

[B5] Chen L. Mathema B. Chavda K. D. DeLeo F. R. Bonomo R. A. Kreiswirth B. N. (2014). Carbapenemase-producing *Klebsiella pneumoniae*: molecular and genetic decoding. Trends Microbiol. 22, 686–696. 10.1016/j.tim.2014.09.003 25304194 PMC4365952

[B6] Clancy C. J. Chen L. Shields R. K. Zhao Y. Cheng S. Chavda K. D.. (2013). Epidemiology and molecular characterization of bacteremia due to carbapenem-resistant *Klebsiella pneumoniae* in transplant recipients. Am. J. Transplant. 13, 2619–2633. 10.1111/ajt.12424 24011185 PMC3955300

[B7] Coil D. Jospin G. Darling A. E. (2015). A5-miseq: an updated pipeline to assemble microbial genomes from Illumina MiSeq data. Bioinformatics. 31, 587–589. 10.1093/bioinformatics/btu661 25338718

[B8] Cuzon G. Naas T. Nordmann P. (2011). Functional characterization of Tn4401, a Tn3-based transposon involved in blaKPC gene mobilization. Antimicrob. Agents Chemother. 55, 5370–5373. 10.1128/AAC.05202-11 21844325 PMC3195030

[B9] Cuzon G. Naas T. Truong H. Villegas M. V. Wisell K. T. Carmeli Y.. (2010). Worldwide diversity of *Klebsiella pneumoniae* that produce beta-lactamase blaKPC-2 gene. Emerg. Infect. Dis. 16, 1349–1356. 10.3201/eid1609.091389 20735917 PMC3294963

[B10] De Souza R. C. Dabul A. N. G. Boralli C. M. D. S. Zuvanov L. da Cunha Camargo I. L. B. (2019). Dissemination of blaKPC-2 in an NTEKPC by an IncX5 plasmid. Plasmid 106:102446. 10.1016/j.plasmid.2019.102446 31669115

[B11] Dong N. Zhang R. Liu L. Li R. Lin D. Chan E.-W. C.. (2018). Genome analysis of clinical multilocus sequence Type 11 *Klebsiella pneumoniae* from China. Microb Genom. 4:e000149. 10.1099/mgen.0.000149 29424684 PMC5857376

[B12] Eltai N. O. Kelly B. Al-Mana H. A. Ibrahim E. B. Yassine H. M. Al Thani A.. (2020). Identification of mcr-8 in clinical isolates from qatar and evaluation of their antimicrobial profiles. Front Microbiol. 11:1954. 10.3389/fmicb.2020.01954 32983006 PMC7476323

[B13] Gandra S. Burnham C-. A. D. (2020). Carbapenem-resistant Enterobacterales in the USA. Lancet. Infect. Dis. 20, 637–639. 10.1016/S1473-3099(20)30066-9 32151330

[B14] Institute Pasteur (2025). MLST and whole genome MLST databases. Available online at: https://bigsdb.pasteur.fr/cgi-bin/bigsdb/bigsdb.pl?db=pubmlst_klebsiella_seqdef (accessed May 10, 2025).

[B15] Jati A. P. Sola-Campoy P. J. Bosch T. Schouls L. M. Hendrickx A. P. A. Bautista V.. (2023). Widespread detection of yersiniabactin gene cluster and its encoding integrative conjugative elements (ICEKp) among nonoutbreak OXA-48-producing *Klebsiella pneumoniae* clinical isolates from Spain and the Netherlands. Microbiol. Spectr. 11:e0471622. 10.1128/spectrum.04716-22 37310221 PMC10434048

[B16] Johnson C. M. Grossman A. D. (2015). Integrative and conjugative elements (ICEs): what they do and how they work. Annu. Rev. Genet. 49, 577–601. 10.1146/annurev-genet-112414-055018 26473380 PMC5180612

[B17] Karaiskos I. Daikos G. L. Gkoufa A. Adamis G. Stefos A. Symbardi S.. (2021). Ceftazidime/avibactam in the era of carbapenemase-producing *Klebsiella pneumoniae*: experience from a national registry study. J. Antimicrob. Chemother. 76, 775–783. 10.1093/jac/dkaa503 33249436

[B18] Karampatakis T. Tsergouli K. Behzadi P. (2023). Carbapenem-resistant *Klebsiella pneumoniae*: virulence factors, molecular epidemiology and latest updates in treatment options. Antibiotics. 12:234. 10.3390/antibiotics12020234 36830145 PMC9952820

[B19] Kitchel B. Rasheed J. K. Patel J. B. Srinivasan A. Navon-Venezia S. Carmeli Y.. (2009). Molecular epidemiology of KPC-producing *Klebsiella pneumoniae* isolates in the United States: clonal expansion of multilocus sequence type 258. Antimicrob. Agents Chemother. 53, 3365–3370. 10.1128/AAC.00126-09 19506063 PMC2715580

[B20] Koren S. Walenz B. P. Berlin K. Miller J. R. Bergman N. H. Phillippy A. M. (2017). Canu: scalable and accurate long-read assembly via adaptive k-mer weighting and repeat separation. Genome Res. 27, 722–736. 10.1101/gr.215087.116 28298431 PMC5411767

[B21] Lindgreen S. (2012). AdapterRemoval: easy cleaning of next-generation sequencing reads. BMC Res. Notes. 5:337. 10.1186/1756-0500-5-337 22748135 PMC3532080

[B22] Luo R. Liu B. Xie Y. Li Z. Huang W. Yuan J.. (2012). SOAPdenovo2: an empirically improved memory-efficient short-read *de novo* assembler. Gigascience 1:18. 10.1186/2047-217X-1-18 23587118 PMC3626529

[B23] MacKenzie F. M. Forbes K. J. Dorai-John T. Amyes S. G. Gould I. M. (1997). Emergence of a carbapenem-resistant *Klebsiella pneumoniae*. Lancet. 350:783. 10.1016/S0140-6736(05)62567-6 9298003

[B24] Munoz-Price L. S. Poirel L. Bonomo R. A. Schwaber M. J. Daikos G. L. Cormican M.. (2013). Clinical epidemiology of the global expansion of *Klebsiella pneumoniae* carbapenemases. Lancet Infect Dis. 13, 785–796. 10.1016/S1473-3099(13)70190-7 23969216 PMC4673667

[B25] Naas T. Oueslati S. Bonnin R. A. Dabos M. L. Zavala A. Dortet L.. (2017). Beta-lactamase database (BLDB) - structure and function. J. Enzyme Inhib. Med. Chem. 32, 917–919. 10.1080/14756366.2017.1344235 28719998 PMC6445328

[B26] Nordmann P. Cuzon G. Naas T. (2009). The real threat of *Klebsiella pneumoniae* carbapenemase-producing bacteria. Lancet Infect. Dis. 9, 228–236. 10.1016/S1473-3099(09)70054-4 19324295

[B27] Octavia S. Kalisvar M. Venkatachalam I. Ng O. T. Xu W. Sridatta P. S. R.. (2019). *Klebsiella pneumoniae* and *Klebsiella quasipneumoniae* define the population structure of blaKPC-2*Klebsiella*: a 5 year retrospective genomic study in Singapore. J. Antimicrob. Chemother. 74, 3205–3210. 10.1093/jac/dkz332 31504571

[B28] Podschun R. Ullmann U. (1998). Klebsiella spp. as nosocomial pathogens: epidemiology, taxonomy, typing methods, and pathogenicity factors. Clin Microbiol Rev. 11, 589–603. 10.1128/CMR.11.4.589 9767057 PMC88898

[B29] Pu D. Zhao J. Chang K. Zhuo X. Cao B. (2023). “Superbugs” with hypervirulence and carbapenem resistance in *Klebsiella pneumoniae*: the rise of such emerging nosocomial pathogens in China. Sci. Bull. 68, 2658–2670. 10.1016/j.scib.2023.09.040 37821268

[B30] Qi Y. Wei Z. Ji S. Du X. Shen P. Yu Y. (2011). ST11, the dominant clone of KPC-producing *Klebsiella pneumoniae* in China. J. Antimicrob. Chemother. 66, 307–312. 10.1093/jac/dkq431 21131324

[B31] Sader H. S. Castanheira M. Shortridge D. Mendes R. E. Flamm R. K. (2017). Antimicrobial activity of ceftazidime-avibactam tested against multidrug-resistant Enterobacteriaceae and Pseudomonas aeruginosa isolates from U.S. medical centers, 2013 to 2016. Antimicrob. Agents Chemother. 61, e01045–17. 10.1128/AAC.01045-17 28827415 PMC5655084

[B32] Seemann T. (2014). Prokka: rapid prokaryotic genome annotation. Bioinformatics 30, 2068–2069. 10.1093/bioinformatics/btu153 24642063

[B33] Seki L. M. Pereira P. S. de Souza M. d. P. A. H. de Conceição M. S. Marques E. A. Porto C. O.. (2011). Molecular epidemiology of KPC-2- producing *Klebsiella pneumoniae* isolates in Brazil: the predominance of sequence type 437. Diagn. Microbiol. Infect. Dis. 70, 274–277. 10.1016/j.diagmicrobio.2011.01.006 21397425

[B34] Smith Moland E. Hanson N. D. Herrera V. L. Black J. A. Lockhart T. J. Hossain A.. (2003). Plasmid-mediated, carbapenem-hydrolysing beta-lactamase, KPC-2, in *Klebsiella pneumoniae* isolates. J. Antimicrob. Chemother. 51, 711–714. 10.1093/jac/dkg124 12615876

[B35] Walther-Rasmussen J. Høiby N. (2007). Class A carbapenemases. J. Antimicrob. Chemother. 60, 470–482. 10.1093/jac/dkm226 17595289

[B36] Wang G. Huang T. Surendraiah P. K. Wang K. Komal R. Zhuge J.. (2013). CTX-M β-lactamase-producing *Klebsiella pneumoniae* in suburban New York City, New York, USA. Emerg Infect Dis. 19, 1803–1810. 10.3201/eid1911.121470 24188126 PMC3837662

[B37] Woodford N. Tierno P. M. Young K. Jr Tysall L. Palepou M. F. Ward E.. (2004). Outbreak of *Klebsiella pneumoniae* producing a new carbapenem-hydrolyzing class A beta-lactamase, KPC-3, in a New York medical center. Antimicrob. Agents Chemother. 48, 4793–4799. 10.1128/AAC.48.12.4793-4799.2004 15561858 PMC529220

[B38] Xu L. Sun X. Ma X. (2017). Systematic review and meta-analysis of mortality of patients infected with carbapenem-resistant *Klebsiella pneumoniae*. Ann. Clin. Microbiol. Antimicrob. 16:18. 10.1186/s12941-017-0191-3 28356109 PMC5371217

[B39] Yigit H. Queenan A. M. Anderson G. J. Domenech-Sanchez A. Biddle J. W. Steward C. D.. (2001). Novel carbapenem-hydrolyzing beta-lactamase, KPC-1, from a carbapenem-resistant strain of *Klebsiella pneumoniae*. Antimicrob. Agents Chemother. 45, 1151–1161. 10.1128/AAC.45.4.1151-1161.2001 11257029 PMC90438

[B40] Zhang R. Liu L. Zhou H. Chan E. W. Li J. Fang Y.. (2017). Nationwide Surveillance of Clinical Carbapenem-resistant Enterobacteriaceae (CRE) Strains in China. EBioMedicine. 19, 98–106. 10.1016/j.ebiom.2017.04.032 28479289 PMC5440625

[B41] Zhang Y. Wang Q. Yin Y. Chen H Jin L Gu B.. (2018). Epidemiology of carbapenem-resistant enterobacteriaceae infections: report from the China CRE network. Antimicrob. Agents Chemother. 62, e01882–17. 10.1128/AAC.01882-17 29203488 PMC5786810

[B42] Zheng S. H. Cao S. J. Xu H. Feng D. Wan L. P. Wang G. J.. (2018). Risk factors, outcomes and genotypes of carbapenem-nonsusceptible *Klebsiella pneumoniae* bloodstream infection: a three-year retrospective study in a large tertiary hospital in Northern China. Infect. Dis. 50, 443–451. 10.1080/23744235.2017.1421772 29303020

