# Supplementary material for: Dissemination of KPC-2-producing carbapenem-resistant Klebsiella pneumoniae ST792 in Southern China
Source: Front Microbiol. 2025 Jun 6;16:1580739. doi: 10.3389/fmicb.2025.1580739 (PMC12179200; doi:10.3389/fmicb.2025.1580739)
Supplement: Supplementary file 4 [file Data_Sheet_1.docx]

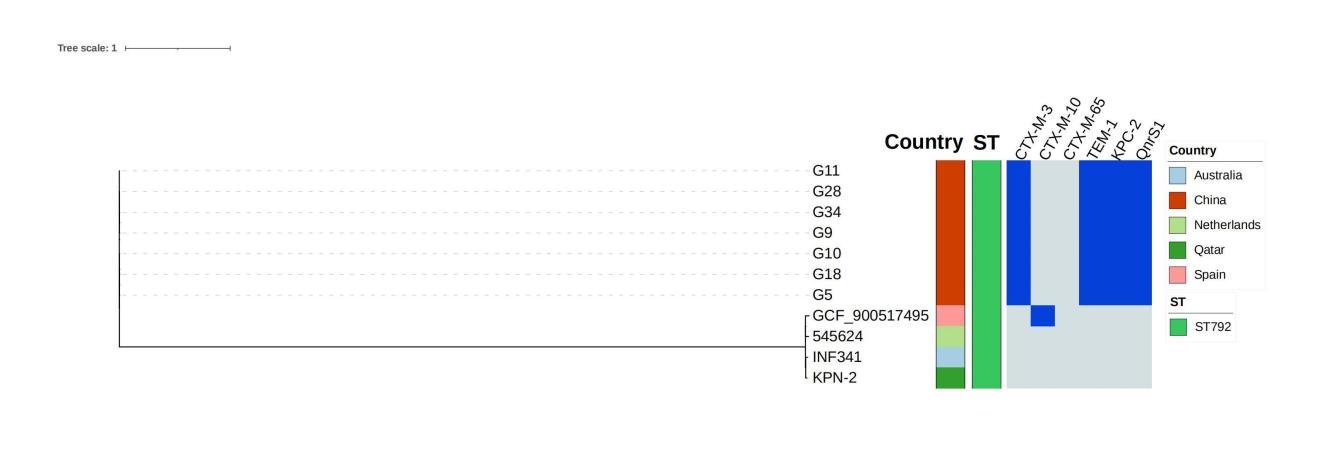
**Supplementary Figures**

Supplementary Figure 1: Core-genome phylogenetic tree of 4 *K. pneumoniae* ST792 genomes from the NCBI RefSeq database and 7 KPC-2-producing strains isolated during a nosocomial dissemination at a tertiary hospital in Ganzhou, Jiangxi Province, China.


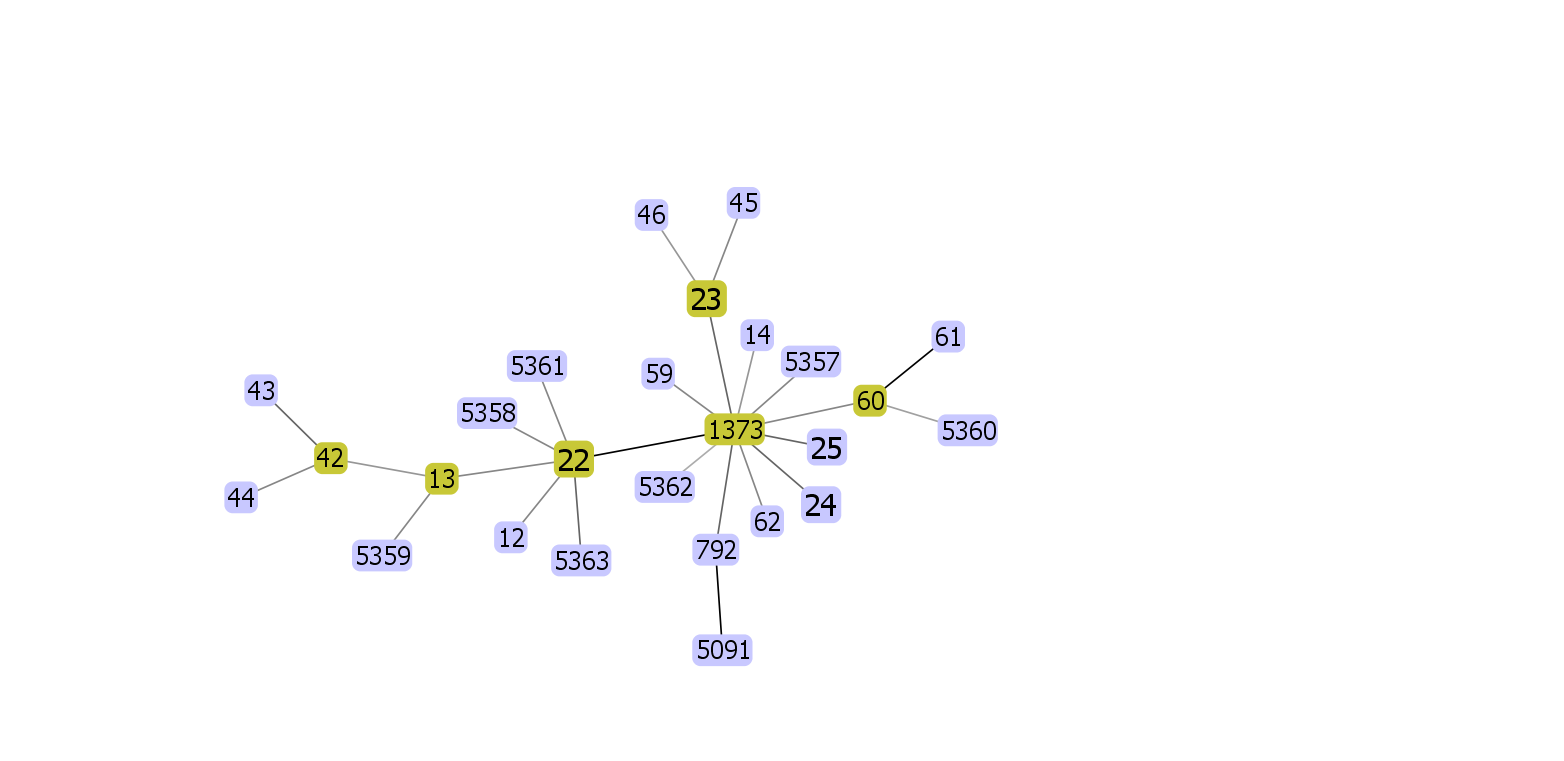


Supplementary Figure 2: Minimum spanning tree of *K. pneumoniae* ST792 and other sequence types (STs) based on MLST allelic profiles, generated using PhyloViz with the goeBURST algorithm.
